# Differential Pattern of Circulating MicroRNA Expression in Patients with Intracranial Atherosclerosis

**DOI:** 10.3390/biomedicines13020514

**Published:** 2025-02-19

**Authors:** Marine M. Tanashyan, Anton A. Raskurazhev, Alla A. Shabalina, Andrey S. Mazur, Vladislav A. Annushkin, Polina I. Kuznetsova, Sergey N. Illarioshkin, Mikhail A. Piradov

**Affiliations:** Research Center of Neurology, Moscow 125367, Russia; m_tanashyan2004@mail.ru (M.M.T.); raskurazhev@neurology.ru (A.A.R.); mazur@neurology.ru (A.S.M.); annushkin@neurology.ru (V.A.A.); snillario@gmail.com (S.N.I.); dir@neurology.ru (M.A.P.)

**Keywords:** microRNA, intracranial atherosclerosis, biomarkers, stroke

## Abstract

**Background:** Intracranial atherosclerosis (ICAS) is a major cause of ischemic stroke, yet fundamental studies regarding epigenetic regulation of ICAS are lacking. We hypothesized that, due to anatomical and/or functional differences, extracranial atherosclerosis is distinct from ICAS, which may explain the clinical variability as well. **Methods:** We chose a number of miRNAs involved in various steps of atherogenesis (namely, miR-712/205-5p/-3p, miR-106b-3p/-5p, miR-146a-3p/-5p, miR-100-3p/miR-5p, miR-200c-3p/-5p, miR-532-3p/-5p, and miR-126-3p/-5p) and examined their plasma levels in a cohort of patients with carotid stenosis > 50% (n = 35, mean age: 65 years, 54% male; 12 patients had ICAS). **Results:** A differential pattern of circulating miR expression was found in ICAS patients: there was an overexpression of miR-712/205-5p, miR-106b-5p, miR-146a-5p, miR-200c-5p, miR-532-3p, and miR-126-3p. The following miRs were underexpressed in intracranial atherosclerosis—miR-712/205-3p and miR-100-3p. These changes represent a plethora of atherogenic mechanisms: smooth muscle cell migration (miR-712/205, miR-532), foam cell formation (miR-106b, miR-146a), endothelial dysfunction (miR-200c), low-density lipoprotein-induced vascular damage (miR-100), and leukocyte recruitment (miR-126). In symptomatic ICAS patients, we observed a statistically significant upregulation of miR-712/205-3p and miR-146a-5p. **Conclusions:** Overall, the findings of our pilot study revealed several new and interesting associations: (1) intracranial atherosclerosis seems to have a different epigenetic profile (regarding circulating microRNA expression) than isolated extracranial vessel involvement; (2) ischemic stroke in ICAS may be potentiated by other pathophysiologic mechanisms than in extracranial-only atherosclerosis (ECAS). Certain miRs (e.g., miR-712/205) seem to have a larger impact on ICAS than on extracranial atherosclerosis; this may be potentially linked to difference between extra- and intracranial artery morphology and physiology, and/or may lead to the said differences. This underscores the importance of making a distinction in future epigenetic studies between ECAS and ICAS, as the mechanisms of atherogenesis are likely to vary.

## 1. Introduction

Intracranial atherosclerosis (ICAS) is a major cause of ischemic stroke via branch and in situ thrombotic occlusion [1]; it is the underlying cause of ischemic stroke in approximately 40% of Asian and 30% of Black patients, but only 10% of White patients [2]. The term ICAS comprises cerebral arteries distal to the internal carotid arteries (ICAs) after they enter the petrosal bone (C2 segment) and the vertebral arteries after they enter the foramen magnum and pierce the dura mater (V4 segment) [3]. Though divided by a somewhat quasi-artificial boundary (i.e., cranial cavity), there exist several crucial differences between extra- and intracranial atherosclerosis—the structure of the vessel wall, lumen diameter, metabolism, and certain hemodynamic properties (e.g., branching, arterial wall shear stress, blood flow velocity, etc.) [4].

ICAS usually manifests two decades later on average than its extracranial counterpart [5], which may (in part) be explained by a relatively more stable plaque phenotype in the former [6]. In ICAS, there are multiple stroke mechanisms, which may vary depending on the site and affected artery: artery-to-artery embolization, impaired distal perfusion, perforator disease, in situ thrombosis, and mixed pattern [7]. Recurrent stroke rates in ICAS are also much higher than in other stroke subtypes, reaching 20% in the first year in certain populations [8].

The intracranial artery morphology is distinct from the extracranial artery morphology, as it lacks an external elastic lamina (which maintains the elasticity of the arterial wall), has less smooth muscle cells (SMC) in a thinner media, and the internal elastic lamina is thicker; the latter may play a role in preventing SMC migration to the intima, an important step in atherosclerosis initiation [9]. Another significant difference is the rare presence of vasa vasorum in the adventitia (heavily involved in atherogenesis by inducing inflammation), supporting the theory of luminal diffusion and partly explaining a more stable phenotype of ICAS plaques [10]. In addition, intracranial arteries give rise to a large number of perforating branches which originate at a right angle, have a tortuous course and take part in collateral circulation [11].

Apart from anatomical distinctions, intracranial vessels present with a larger antioxidant response compared with extracranial vessels (e.g., significantly greater activity of the free radical scavenger Mn-SOD), which may contribute to later disease onset. Another possible factor influencing a differential pattern of ICAS development may be a less pronounced reaction of the vessel wall to hypercholesterolemia, possibly due to more tight endothelial junctions and/or the presence of structures comprising the blood–brain barrier [12]. D’Armiento et al. showed that the antioxidant properties of intracranial arteries markedly decrease with advanced age and this coincides with a rapid acceleration of atherosclerosis in the intracranial arteries of elderly subjects [13].

Atherosclerosis is a fundamental pathologic process which underlies a vast majority of morbidity and mortality in the world, despite proven preventive and treatment strategies. The search for clear and precise biomarkers in this field is of paramount importance as it provides guidance for the necessary risk stratification and prognosis, as well as therapeutic response. However, as shown in [14], there is scarce hope that a single valid biomarker could be found for such a complex pathology; instead, we should rely on multiple biomarker profiling within a personalized paradigm.

Atherogenesis is a complex pathology, which involves multiple epigenetic mechanisms, including microRNAs (miRNAs)—short, single-stranded, and non-protein coding RNAs, post-transcriptionally regulating the expressions of genes via targeting the 3′ untranslated regions in mRNAs [15]. The important role of miRNAs, as vigorous regulators of many biological processes, including cell growth, proliferation, differentiation, migration, senescence, apoptosis, and angiogenesis, is widely recognized [16]. Circulating miRNAs are potential mediators of almost all crucial steps of atherosclerosis progression. Fundamental differences between ICAS and extracranial atherosclerosis may arise due to anatomical and functional variances or disparities in risk factor distribution, all of which could be mediated by miRNA.

The existing data on miRNA involvement in ICAS development are scarce, so the main goal of this pilot study was to elucidate whether the profile of circulating miRNA expression (involved in various steps of atherogenesis) in ICAS is distinct from that of isolated extracranial vessel involvement. Previously, we found a number of circulating miRNAs relevant for extracranial atherosclerosis; thus, we utilized these findings to select appropriate miRNAs for the present study [17].

## 2. Materials and Methods

### 2.1. Study Population

We included in this cross-sectional study 35 patients (mean age—65 years, 54% male) with hemodynamically significant extracranial carotid atherosclerosis, verified via ultrasound examination. Based on MRI and/or CT imaging, 12 patients were found to have intracranial vessel atherosclerosis (83% male). We excluded patients with decompensated somatic (e.g., cardiovascular, renal, hepatic) disease, a history or current signs of neoplasm, concurrent infectious, autoimmune, rheumatic disorder, and less than 3 months after major surgery. All patients provided informed consent.

All patients underwent a thorough clinical and neurological examination, routine blood analyses (including low-density lipoprotein cholesterol [LDL-C] and total cholesterol [TC]), and ultrasound examination of the extracranial carotid arteries (with luminal stenosis measured according to the NASCET criteria to exclude carotid atherosclerosis) (Table 1).

### 2.2. Laboratory Analysis

RNA (including microRNA) was isolated from 200 µL of serum using the miRNeasy Serum/Plasma Advanced Kit—microRNA Isolation (Qiagen, Hilden, Germany) in accordance with the manufacturer’s protocol. The RNA concentration was evaluated using a Qubit 4 fluorimeter (Thermo Fisher Scientific, Waltham, MA, USA). Exogenous synthetic cel-miR-39-3p (0.2 nM long mi-croRNA *C. elegans*) was used to normalize microRNA levels (recommended by manufacturer). Mir-16-5p was chosen as an endogenous normalizer of circulating microRNA expression due to its relative stability in serum/plasma shown by various studies and its widespread use as a reference in the association of circulating microRNAs with various pathologies [18].

RNA samples (final volume 20 µL) were stored at a temperature of −80 °C until the stage of cDNA synthesis in the reverse transcription reaction.

For reverse transcription and determination of microRNA transcripts, sets of «AL-MIR» reagents (Algimed Techno RT-PCR, Minsk, Belarus) containing optimized en-zyme mixtures for reverse transcription and PCR. Optimized solutions of oligonucleotides for the analysis of a specific miRNA molecule were used: an RT-primer, a pair of PCR primers, and a fluorescently labeled probe for amplification detection. The volume of the microRNA sample for cDNA synthesis was 2 µL, in accordance with the manufacturer’s instructions.

Quantitative determination of microRNA transcripts was performed using real-time PCR using the CFX96 C1000 Touch amplifier (BioRad, Hercules, CA, USA) according to the following program: activation of the enzyme for 20 s at 95 °C, 45 cycles, denaturation for 1 s at 95 °C, and annealing/elongation for 20 s at 60 °C. The relative microRNA level was calculated using the 2^−ΔΔCt^ method using the appropriate CFX Manager 3.1 software [17].

### 2.3. Statistical Analysis

Statistical analysis was performed in R programming language (version 4.4.1) via RStudio interface (version 1.4.1717), with the following downloadable packages: ‘tidyverse’, ‘finalfit’, ‘heatmaply’, ‘gtsummary’, and ‘randomForest’. We utilized descriptive statistics with median (interquartile range) for continuous variables (e.g., age) and frequency/proportion for discrete variables (e.g., gender). Between-group analysis was performed with Pearson’s Chi-squared test (for proportions) and Wilcoxon rank sum test (for continuous variables). Regression analysis included building univariate logistic regression models (estimating odds ratios and 95% confidence intervals) and multivariate regression with random forest (number of trees: 1000, number of variables tried at each split: 4; estimating mean decrease in accuracy and mean decrease in Gini coefficient as measures of variable importance). All tests were two-sided and the alpha-level was 0.1.

## 3. Results

**Figure 1:**
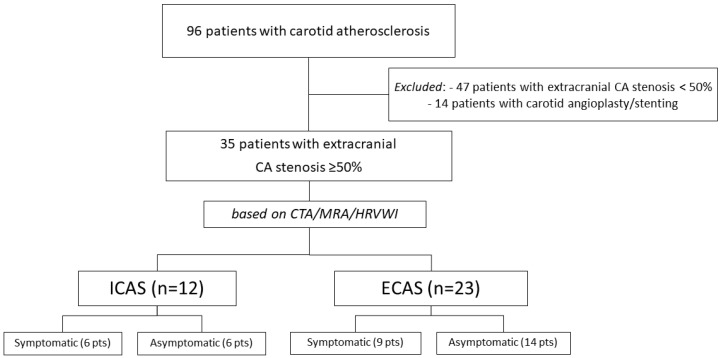
Flow-chart of patients included in this study. ICAS—intracranial atherosclerosis, ECAS—extracranial only atherosclerosis, CTA—CT-angiography, MRA—magnetic resonance angiography, HRVWI—high-resolution vessel wall imaging.

We included 35 patients in this small pilot case study (12 with ICAS + extracranial CA and 23 with extracranial CA only [ECAS], Figure 1). The main findings are presented in Table 2.

All patients had arterial hypertension as a comorbidity. No statistically significant differences were observed between groups with ICAS and extracranial CA regarding stroke occurrence, age, smoking status, diabetes frequency, and basic laboratory parameters (including the levels of LDL). A male predominance was found in the ICAS group, which reached the level of statistical significance (*p* = 0.033). This may be (at least in part) attributed to a higher burden of atherosclerotic plaque in men overall [19]. There were no relevant clinical differences (in terms of symptoms, focal signs, etc.) between symptomatic patients with and without ICAS.

A differential pattern of circulating miR expression was found in ICAS patients: there was overexpression of miR-712/205-5p, miR-106b-5p, miR-146a-5p, miR-200c-5p, miR-532-3p, and miR-126-3p; and the following miRs were underexpressed in intracranial atherosclerosis—miR-712/205-3p and miR-100-3p. Interestingly, two strands of the same miR-712/205 (namely, 5p and 3p) were differentially expressed in ICAS patients, which is somewhat unusual, given the fact that 3p/5p strands are on the whole very closely correlated.

We performed a univariate logistic regression to elucidate statistically significant associations of clinical and laboratory characteristics with ICAS (Supplementary Table S1). Then (considering for a small sample size), we built a multivariate logistic regression model using random forest analysis (Supplementary Figure S1) and analyzed each variable importance, both using the mean decrease in accuracy and the mean decrease in Gini coefficient. The most important was the expression levels of miRs (miR-146a-5p, miR-200c-5p, miR-712-3p, and miR-106b-5p).

We then addressed smoking as major risk factor for atherosclerosis, and, in order to check if this factor accounted for observed variation in miR expression, a logistic regression was performed (Supplementary Table S2) which found that only two factors were statistically associated with smoking—age and male gender (*p* < 0.05). A separate analysis of patients with extracranial atherosclerosis-only involvement (ECAS) (Table 3) also did not show any statistically significant differences in the expression levels of studied miRs.

In order to elucidate the possible involvement of epigenetic regulation of ICAS via miR and stroke occurrence, we examined the whole cohort of ICAS patients (n = 12) and their symptomatic (i.e., prior ipsilateral ischemic stroke) status. A brief summary of ICAS patient characteristics is presented in Table 4.

**Figure 2:**
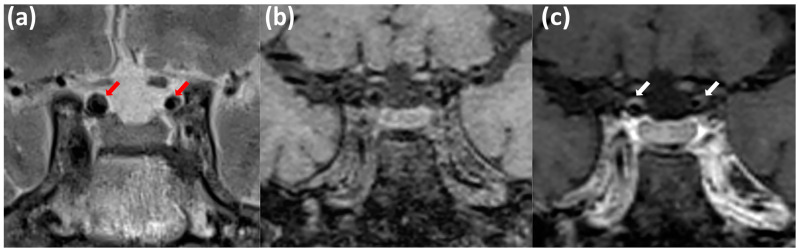
Patient ID#2. MRI signs of intracranial atherosclerosis of the brachiocephalic arteries: uneven stenosis of both internal carotid arteries (ICA) at the intracranial level, with signs of an eccentric pattern of contrast enhancement. MRI in T2 mode with high resolution (T2-HR) in the coronary (**a**), MRI T1 db f/s, before (**b**) and after (**c**) intravenous contrast injection with high-spatial-resolution T1-weighed imaging and suppression of the signal from bloodstream and fat. Red arrows—intracranial atherosclerotic plaque; white arrows—contrast enhancement in the vessel wall.

**Figure 3:**
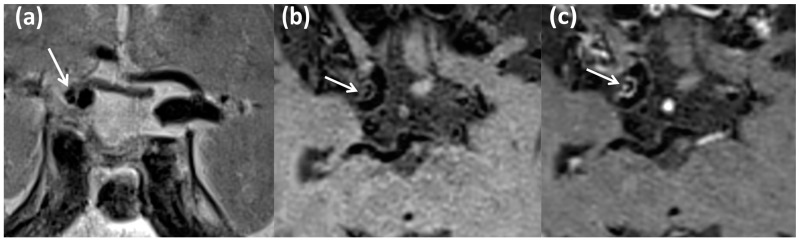
Patient ID#1. The MRI data correspond to stenosis of the left internal carotid artery (60–65%) with signs of an eccentric pattern of contrast enhancement. MRI in T2 mode with high resolution (T2-HR) in the coronary plane (**a**), MRI T1 db f/s, before (**b**) and after (**c**) intravenous contrast injection with high-spatial-resolution T1-weighed imaging and suppression of the signal from bloodstream and fat. White arrows—intracranial atherosclerotic plaque.

As examples, we show in Figure 2 and Figure 3 the MRI pattern characteristic of intracranial atherosclerotic lesions.

**Figure 4:**
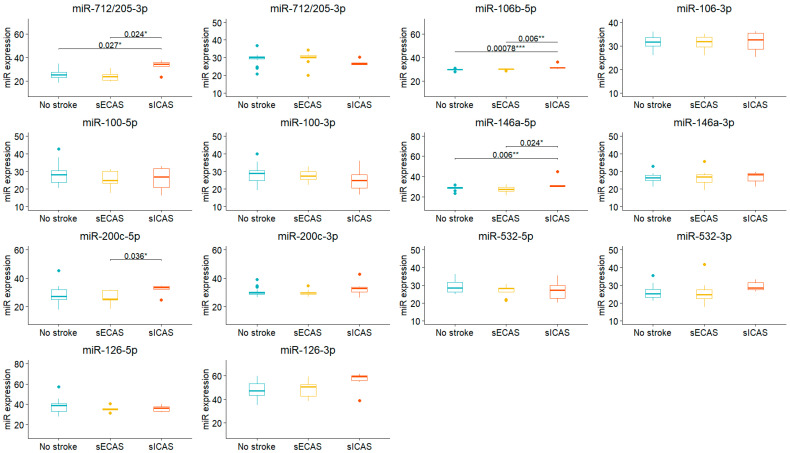
Box-plot showing miR expression in symptomatic patients with ICAS (sICAS), ECAS (sECAS), and patients without prior stroke. Shown are only significant pairwise differences (Holm–Bonferroni correction for multiple comparisons, *p* < 0.05). * *p* < 0.05; ** *p* < 0.01; *** *p* < 0.001.

We then analyzed possible differential miRNA expression in symptomatic (ICAS and ECAS) patients between each other, and versus patients without stroke (Figure 4).

In symptomatic ICAS patients, we observed a statistically significant upregulation of miR-712/205-3p and miR-146a-5p, both versus ECAS patients with prior stroke and the asymptomatic group. Another miRNA that was overexpressed in ICAS stroke patients was miR-200c-5p, compared to patients with isolated extracranial artery involvement. Circulating levels of miR-106b-5p were also statistically significant higher in patients with ICAS-associated stroke than in the other two groups of patients.

## 4. Discussion

Intracranial atherosclerosis (ICAS) is a major cerebrovascular pathology which nevertheless seems to be overshadowed by its more studied extracranial counterpart. Epigenetic mechanisms involving microRNAs play a critical role in all steps of atherogenesis, yet the degree of their engagement may be different depending on the location. We have shown in this pilot study that the expression of circulating miRs associated with atherosclerosis varies in patients with ICAS compared to patients who have only extracranial vessel involvement. Moreover, differential miR expression was found depending on stroke occurrence in patients with ICAS.

We performed a literature review to elucidate possible already known associations of miRNA expression and ICAS. We employed the following search query to identify relevant manuscripts from PubMed database: ((intracranial atherosclerosis) OR (intracranial atherosclerotic disease)) AND ((microRNA) OR (miRNA)). A total of 57 publications was identified (dating from 2012 to 2024). Of them, we included 16 relevant studies; they are briefly described in Table 5. Among the reasons for exclusion were interventional studies without focus on atherosclerosis (n = 9), manuscripts concerning dementia and/or vascular cognitive impairment (n = 17), no or scarce mention of atherosclerosis (n = 10), and review articles not relevant to the topic (n = 5). Only two studies (Jeong H. et al., 2017 [20]; Jiang H. et al., 2019 [21]) explicitly recruited patients with ICAS: both showed differential expression of a number of miRs, but the first showed this in patients compared to control, while the second found this with respect to responses to intensive medical management of ICAS. miR-212 was shown in the study by Jeong H. et al. (2017) [20] to be a promising biomarker for ICAS, a finding which may be corroborated using in vivo studies. Downregulating SIRT1, miR-212 suppresses cholesterol efflux, enhancing foam cell formation, a crucial step of atherogenesis [22].

A number of miRs appear in several of these studies repeatedly, underlining their possible significant involvement in atherogenesis (including ICAS). For example, miR-126 is a predominantly endothelial-expressed miR, which is essential in maintaining vascular integrity and endothelial homeostasis [23]. Gao J. et al. (2019) [24], in a study of patients with large-artery atherosclerosis (both symptomatic and asymptomatic), found that the plasma expression of miR-126 is minimally affected by previous cerebral infarction and is correlated to the severity of atherosclerosis (identified as the number of affected arteries).

MiR-146a—another microRNA involved in endothelial dysfunction and inflammation—was found to be highly expressed in patients with vascular dementia (Dong et al., 2015 [25]), which is increasingly associated with ICAS [26]. Interestingly, in a study by Zhong et al. (2016) [27], a complex interplay was observed between miR-146a and the ApoEε4 genotype, with an increased risk of atherosclerotic cerebral infarction in patients with a reduced expression of miR-146a. This may suggest an atheroprotective role of miR-146a attributed to the suppression of pro-inflammatory markers (mainly NF-κB signaling) and endothelial activation. In our study, ICAS patients had upregulated levels of miR-146a-5p, indicating a possible protective phenotype, which was further supported using in vivo studies, demonstrating its role in inhibition of foam cell formation [28]. Paradoxically, though, miR-146a levels are elevated in atherosclerotic plaques [29]. Together with our findings regarding its overexpression in symptomatic stroke patients, this may indicate a potential feedback loop, where high levels of inflammatory (or in the case of stroke, neuroinflammatory) markers lead to the upregulation of miR-146a, and not vice versa.

Higher levels of miR-155 in endothelial microvesicles were reported by Zhang et al. (2020) [30] in ischemic stroke patients, particularly of the atherothrombotic and cardioembolic subtypes. A review by Volný et al. (2015) [31] suggests that it may play a proatherogenic role, involved in inflammation and macrophage accumulation.

It is important to elucidate a number of distinct features which differentiate ICAS from extracranial atherosclerosis and may distinguish ICAS as a separate pathological phenomenon (with a separate microRNA expression profile). Morphologically, in intracranial arteries there is a well-developed internal elastic lamina in the absence of an external elastic plate, a deficiency of elastic fibers in the medial layer, thin adventitia, and a small amount of vasa vasorum, which play an important role in the process of atherogenesis. A number of anatomical features—a large number of tortuosities, a 90° angle of perforant arteries—all contribute to hemodynamic changes in blood flow and shear stress fluctuations. These factors create favorable conditions for the formation and progression of atherosclerotic plaque. We hypothesized that several microRNAs may be relevant to these atherogenic factors, including miR-712/205, miR-532, miR-106b, and miR-200c (not identified in the literature search).

Disturbed flow in intracranial arteries may be associated with pro-atherogenic changes, including endothelial activation and miR-712/205 overexpression. It has been shown by Son et al. (2013) [32] that miR-712/205 downregulates the tissue inhibitor of metalloproteinase-3 (TIMP3) expression, which in turn activates the downstream matrix metalloproteinases (MMPs) and stimulates pro-atherogenic responses and endothelial inflammation and permeability. Moreover, an anti-miR-712/205 treatment resulted in the prevention of atheroma formation in a mouse model of atherosclerosis [33]. A nanoparticle delivery of anti-miR-712 significantly increased the TIMP3 expression in inflamed endothelium [34]. These mechano-receptive miRs may play a greater role in ICAS than in extracranial atherosclerosis due to the angle of perforator origin. MiR-712/205-5p was upregulated in patients with ICAS (30.0 vs. 23.9), partially confirming this suggestion; however, the 3p-strand of miR-712 was downregulated in ICAS patients. This finding may indicate a differential role of this microRNA in cerebral atherosclerosis. Interestingly, miR-712/205 was found to be statistically significantly overexpressed in patients with ICAS and prior stroke, which may corroborate our hypothesis about its predominant impact not only in the development, but also in complications of intracranial atherosclerosis.

Lipid metabolism has also been shown to differentially impact extracranial and intracranial atherosclerosis [35]. This, taken together with the promotion of ox-LDL-induced endothelial damage, may explain the differential expression of several miRs in ICAS patients. For example, miR-200c-5p circulating levels were higher in ICAS patients, while miR-100 was downregulated; the latter finding confirms previously published research [36], which demonstrated that miR-100 may alleviate ox-LDL-induced vascular damage. Promotion of ox-LDL-induced endothelial apoptosis and subsequent atherosclerosis progression may be caused by the upregulation of miRNA-200 via targeting of HDAC4 [37].

The overexpression of miR-532-5p may inhibit the proliferation and migration of VSMCs, a crucial step of atherogenesis [38]. In ox-LDL-induced human brain microvascular endothelial cells, miR-532-5p was downregulated, meaning it could alleviate vascular damage [39]. In our study, miR-532 levels were higher in ICAS patients than in patients with its extracranial counterpart. This may indicate that, ceteris paribus, either miR-532 overexpression is linked to a more favorable profile of intracranial plaques or it has a dual role, promoting ICAS. Given that no differences in miR-532 levels were found depending on stroke occurrence, it seems that the former suggestion is more plausible. One of the targets of miR-532 is programmed cell death protein 4 (PDCD4), a molecule implicated in regulating the proliferation and apoptosis of SMCs, which was found to be highly expressed in the coronary artery tissues [40].

The upregulation of miR-106b-3p in atherogenesis is associated with promoting foam cell formation and inhibiting macrophage cholesterol efflux [41]. A study by Giannella et al. [42] demonstrated that miR-106b-3p is one of the most promising potential markers for the discrimination of plaque composition at the carotid level, which may be relevant for intracranial atherosclerosis. Circulating levels of miR-106b were elevated more than three-fold in patients with atherosclerosis, and were significantly higher in patients with hypercholesterolemia or diabetes in a study by Telkoparan-Akillilar et al. [43]. Conversely, the upregulation of miR-106b-5p has been found to mitigate increased atherogenesis and amplified inflammation induced by linc00657, a long non-coding RNA which is (among other targets) associated with the overexpression of TXNIP, increasing intracellular oxidative stress [44]. Another study provided further insights into the possible atheroprotective role of miR-106b overexpression, which inhibited endothelial cell apoptosis in ox-LDL-induced models of atherosclerosis; the authors suggested that the PI3K/AKT pathway and downregulation of PTEN as a potential target [45].

**Table 5:** A summary of 16 relevant studies of microRNAs in intracranial atherosclerosis.

Study	Patient Population	Data on ICAS	Examined miRs	Relevant Conclusion	Commentary
Long G. et al. (2013) [46]	Stroke patients (n = 197), incl. large-vessel atherosclerosis (n = 51); control (n = 50)	None	Circulating levels of miR-30a, miR-126, and let-7b	Circulating let-7b was lower in patients with large-vessel atherosclerosis than healthy volunteers	Possibly in some patients with LAA stroke could be attributed to ICAS
Dong H. et al. (2015) [25]	Alzheimer’s disease (n = 127), mild cognitive impairment (n = 30), and vascular dementia (VD, n = 30)	None	miR-31, miR-93a, and miR-146a	miR-31, miR-93, and miR-146a levels were significantly higher in the VD patients than in the controls	Some cases of VD could be due to ICAS
Volný O. et al. (2015) [31]	NA (review)	NA	miR-17-92 cluster, miR-126, miR-143/145 cluster, miR-155, miR-21, and miR-221	miR-155 knockout or inhibition in experimental models of atherosclerosis led to reduction of atherosclerotic plaque size and decreased macrophage accumulation; levels of miR-21 and miR-221 were shown to be higher in patients with carotid atherosclerosis; urinary levels of miR-29b were shown to correlate with cIMT in patients with type 2 diabetes mellitus	No division into extra- and intracranial atherosclerosis
Sima X. et al. (2015) [47]	305 patients with intracranial aneurysms and 401 healthy controls	None	rs10877887 and rs13293512 polymorphisms in the promoters of let-7 family	Association of the rs13293512 polymorphism in the promoter region of let-7 with intracranial aneurysms	Data on intracranial vessel wall miRs may be relevant to ICAS study
Zhong H. et al. (2016) [27]	297 patients with atherosclerotic cerebral infarction (ACI) and 300 matched healthy individuals	None	miR-146a expression	The C allele of rs2910164 of the APOE4 gene may reduce miR-146a expression and subsequently weaken anti-inflammatory action in the pathogenesis of ACI; ACI patients with the ApoEε4 allele exhibited reduced miR-146a expression compared with controls	Some patients could have ICAS; possible molecular interplay between Alzheimer’s disease and atherosclerosis
Jeong H. et al. (2017) [20]	36 atherosclerotic patients, who had severe stenosis in the intracranial and extracranial vessels, and 37 non-atherosclerotic patients, who had no stenosis in the evaluated vessels	ICAS was defined as >3 vessels having >50% of stenosis on 11 intracranial vessels (middle cerebral arteries, anterior cerebral arteries, posterior cerebral arteries, intracranial internal carotid arteries, vertebral arteries of both sides, and basilar artery) on time of flight magnetic resonance angiography; extracranial atherosclerosis: >2 vessels having >50% stenotic area on carotid duplex sonography	14 miRNAs (miR-30c, -99b, -152, -181, -212, -222, -301b, -372, -454, -502, -576, -27a, -744, and -888) showed differential expression between non-atherosclerotic and atherosclerotic patients	The study identified miR-212 as a novel marker that enhanced the estimation power of three established cardiovascular risk markers (HbA1c, HDL-C, and lipoprotein(a)) for atherosclerosis presence in ischemic stroke patients.	Here, patients simultaneously had extra- and intracranial atherosclerosis, with very strict inclusion criteria. One of the few studies with ICAS as the main focus
Li Z. et al. (2017) [48]	23 patients with atherosclerotic cerebral infarction and 32 healthy individuals	None	miR-223	Mean methylation levels of a total of nine CpGs of miR-223 promoter were significantly lower in ACI patients than in healthy individuals, and were also significantly lower in individuals with carotid atherosclerosis than those without carotid atherosclerosis	A relevant proportion of patients could have ICAS
Prabhakar P. et al. (2017) [49]	204 patients with VD, 200 control patients	None	plasma miRNA profiling	Plasma miR-409-3p, miR-502-3p, miR-486-5p, and miR-451a could be used to differentiate small vessel VaD patients from healthy controls	Some cases of VD may attributed to ICAS
Gao J. et al. (2019) [24]	LAA stroke patients (n = 57), Atherosclerosis group (n = 41), control group (n = 50)	None	Plasma miR-126 and miR-143	miR-143 and miR-126 might participate in the atherosclerosis process and are minimally affected by cerebral infarction	Possibly some cases with ICAS (not explicitly)
Li J. et al. (2019) [50]	NA (review)	NA	miR-155, miR-27a/b, miR-342-5p, miR-21, miR-124, miR-223, miR-146a, miR-181b, miR-126, miR-143, and let-7b	Described are multiple roles of the mentioned miRs in regulating the progression of atherosclerosis involved with systemic and local inflammatory activities in cerebral arteries	Great review, but most findings are from general atherosclerosis or carotid atherosclerosis studies
Jiang H. et al. (2019) [21]	Patients with severe ICAS (≥70% stenosis) of any intracranial large artery (n = 74): 29 patients had recurrent ischemic events despite intensive medical management during 6-month follow-up (non-responders) and 45 patients had no ischemic events (responders)	All ICAS patients	Exosomal expression of miR-27b-3p, miR-122-5p, miR-16-5p, miR-30c-5p, miR-486-5p, miR-10a-5p, miR-10b-5p, miR-101-3p, miR-24-3p, miR-192-5p, miR-30c-5p, miR-425-5p, and miR-191-5p	This study suggests a specific circulating e-miRNA expression profile that is associated with antiangiogenesis and is a novel biomarker for recurrent ischemic events despite IMM in severe ICAD	Very informative study with a special focus on treatment; a large number of miRs have shown differential expression, possibly a novel target biomarker for therapy modification in resistant ICAS
Xuan J. et al. (2021) [51]	Cerebral atherosclerosis group (n = 52), control group (n = 46)	Possibly, all ICAS patients (due to enrollment criteria)	miR-137	miR-137 expression was decreased in ICAS; miR-137 was independently correlated with the occurrence of cerebrovascular adverse events	Possibly, antiatherogenic factor
Zhang H. et al. (2020) [30]	97 ischemic stroke patients (LAA, n = 35); 76 healthy controls	None	EMVs-miR-155	EMVs and EMVs-miR-155 levels were higher in LAA and cardioembolic stroke subtypes	Possibly, some patients with LAA had ICAS
Yang S. et al. (2022) [52]	Cerebral atherosclerosis patients (stenosis≥ 50%) (n = 46); patients with no AS or vascular stenosis < 50% were included in the control group (n = 46)	ICAS (due to enrollment criteria)	LncRNA SNHG16, miR-30c-5p and SDC2 expression	miR-30c-5p was downregulated in ICAS; downregulating lncRNA SNHG16 inhibits VSMC proliferation and migration in AS by targeting the miR-30c-5p/SDC2 axis	This study examines a novel non-coding RNAs target interplay involved in the crucial aspect of atherosclerosis development—VSMC migration
Jiang H. et al. (2022) [53]	Patients with intracranial aneurysms (n = 58), control group (n = 44)	None	Serum levels of miR-1246	Serum levels of miR-1246 were elevated in intracranial aneurysm patients	Again, data on miR-1246 may be relevant to ICAS, as it is involved in inflammatory response, lipid, and atherosclerotic signaling pathways
Xu J. et al. (2022) [54]	Cerebral atherosclerosis (n = 74), control group (n = 62)	Probably, most ICAS	Serum miR-130a-3p	Cerebral atherosclerosis patients had elevated serum miR-130a-3p compared with controls; high serum miR-130a-3p was independently related with high probability of cerebrovascular events	A prospective study utilizing a novel significant prognostic marker for stroke in ICAS patients

Notes: NA—not applicable; LAA—large-artery atherosclerosis; cIMT—carotid intima-media thickness; ICAS—intracranial atherosclerosis; VSMC—vascular smooth muscle cells; EMV—endothelial microvesicles.

**Figure 5:**
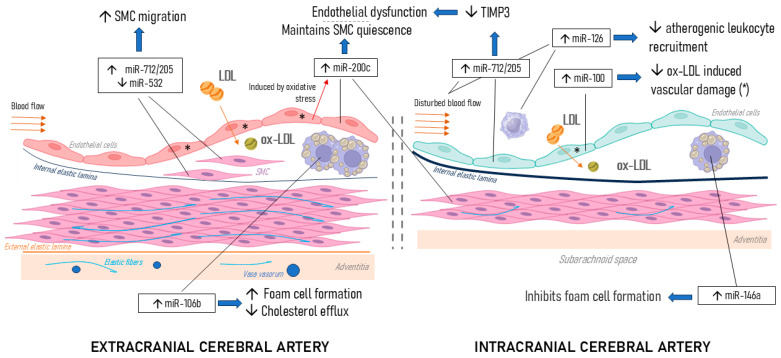
Atherosclerosis-related targets of studied miRs. Shown are cross-sections of extra-(**left**) and intracranial (**right**) arteries with anatomical features characteristic of each level (i.e., a thicker internal elastic lamina, absence of external elastic lamina and vasa vasorum, and low proportion of elastic fibers in intracranial vessels). LDL—low-density lipoprotein; ox-LDL—oxidized LDL; SMCs—smooth muscle cells; TIMP3—metalloproteinase inhibitor 3; * ox-LDL induced vascular damage.

A literature review we performed regarding previously described miRs associated with ICAS revealed a relatively small number of publications which explicitly included patients with this pathology. The possible interplay and target associations between miRs included in our pilot study are summarized in Figure 5.

Overall, the findings of our pilot study reveal several new and interesting associations: (1) intracranial atherosclerosis seems to have a different epigenetic profile (regarding circulating microRMA expression) than isolated extracranial due to vessel involvement; (2) ischemic stroke in ICAS may be potentiated by other pathophysiologic mechanisms than in ECAS. Certain miRs (e.g., miR-712/205) seem to have a greater impact on ICAS than on extracranial atherosclerosis; this may be potentially linked to the difference between extra- and intracranial artery morphology and physiology, and/or may lead to the said differences. A distinct microRNA profile of ICAS may (at least partly) explain clinical differences between the two, i.e., a more stable plaque phenotype, a predisposition to certain stroke subtypes, and greater age of onset. This underscores the importance of making a distinction in future epigenetic studies between ECAS and ICAS, as the mechanisms of atherogenesis are likely to vary. As of potential clinical applications, (1) miRNA may be used as a biomarker to distinguish patients requiring further imaging studies to detect ICAS; (2) miRNA profiling may serve as a surrogate risk marker for the prediction of future cerebrovascular ischemic events and/or the therapeutic response.

## 5. Conclusions

Intracranial atherosclerosis may be a distinct entity, clinically as well as pathologically. The epigenetic regulation of ICAS with respect to microRNA expression is understudied, with most of the research coming from basic atherosclerosis studies. In our pilot study, we found that ICAS has a differentiated profile of miR expression compared with patients exhibiting only extracranial vessel involvement. In ICAS patients we found upregulated miRNAs—miR-712/205-5p, miR-106b-5p, miR-146a-5p, miR-200c-5p, miR-532-3p, and miR-126-3p—involved in the activation of smooth muscle cell migration, foam cell formation, and endothelial dysfunction, while certain miRs (namely miR-712/205-3p and miR-100-3p) were downregulated. Moreover, it seems that ischemic stroke in ICAS occurrence may be associated with a certain degree of abnormal miR signaling; however, experimental and prospective clinical research are desperately required to enhance our understanding of this topic.

### Limitations

This pilot study has a number of limitations which may preclude us from generalizing its results on broader cohorts of patients. First and foremost is the small sample size—this was due (in part) to strict criteria for ICAS diagnostics (all patients underwent MRI/C imaging). Another cofounding factor may be the low prevalence of ICAS in Caucasians—a study comparing cohorts from Oxford and Hong Kong found ICAS prevalence at 43.0% in Chinese patients versus 20.0% in Caucasians [55]. This trend is consistent across multiple studies, indicating that ICAS is more common in younger Asians, while Caucasians show an increased prevalence with age. The male predominance in ICAS patients, while conclusive with previous data, may also indicate selection bias. The choice of studied microRNAs is also a limitation—we could not perform miRNA profiling to identify the most prominent up/downregulated microRNA. Another limitation is the selection bias which may have arisen from recruiting patients from a single center. The overall profile could have been confounded by the exclusion of patients with mild ICAS, as well as an underestimation of the medication effect (e.g., statin therapy and/or antithrombotic drug use). The literature search strategy may also be a limitation, as certain publications where ICAS was not explicitly mentioned could have been omitted.

## Figures and Tables

**Table 1:** Inclusion and exclusion criteria.

Inclusion Criteria	Exclusion Criteria
1 Hemodynamically significant (i.e., >50% luminal stenosis) of extracranial carotid arteries. 2 (For ICAS group) ICAS with stenosis of a major intracranial artery diagnosed using angiogram, MRA, or CTA. 3 Patient understands the purpose and requirements of the study, can make him- or herself understood, and has provided consent.	1 Intracranial arterial stenosis related to arterial dissection, moyamoya disease, or any known infectious or vasculitis disease. 2 Any hemorrhagic infarct or any other intracranial hemorrhage (subarachnoid, subdural, or epidural). 3 Endovascular angioplasty and (or) stenting or major surgery (including open femoral, aortic, or carotid surgery). 4 Intracranial tumor or any sine of neoplasms or intracranial vascular malformation.

**Table 2:** MicroRNA expression and clinical findings depending on ICAS.

Clinical and Laboratory Findings	ICAS		*p*-Value ^2^
	No (n = 23) ^1^	Yes (n = 12) ^1^	
Age, years	67 (62, 72)	65 (59.5, 66.5)	0.16
M	9 (39%)	10 (83%)	0.033
Smoking	10 (43%)	5 (42%)	>0.9
DM	8 (35%)	3 (25%)	0.8
Stroke	9 (39%)	6 (50%)	0.5
Stenosis ^3^,%	75 (70, 80)	70 (70, 90)	>0.9
BMI, kg/m^2^	27.0 (26.0, 34.0)	27.0 (25.7, 28.4)	0.2
RBC, ×10^12^/L	4.60 (4.34, 4.90)	4.76 (4.51, 5.26)	0.12
WBC, ×10^9^/L	7.90 (6.30, 9.40)	8.20 (6.95, 8.55)	>0.9
Platelets, ×10^9^/L	216 (188, 281)	228 (200, 263)	0.7
LDL-C, mmol/L	1.83 (1.34, 2.72)	1.78 (1.20, 2.23)	0.23
miR-712/205-5p	23.9 (21.0, 27.9)	30.0 (24.2, 34.8)	0.034
miR-712/205-3p	30.20 (29.47, 30.49)	27.87 (26.20, 29.81)	0.037
miR-106b-3p	31.48 (28.82, 33.38)	32.26 (27.41, 34.34)	0.5
miR-106b-5p	30.03 (29.68, 30.26)	30.74 (30.24, 31.33)	0.006
miR-146a-3p	26.11 (24.19, 28.25)	27.57 (23.27, 28.68)	0.8
miR-146a-5p	28.58 (26.19, 29.47)	30.06 (29.66, 30.99)	<0.001
miR-100-3p	28.8 (25.4, 31.0)	24.7 (22.2, 29.0)	0.073
miR-100-5p	26.7 (23.3, 29.9)	26.7 (23.1, 32.3)	0.7
miR-200c-3p	29.64 (28.36, 30.01)	30.21 (29.25, 33.33)	0.2
miR-200c-5p	25.3 (24.6, 31.6)	32.5 (25.1, 34.4)	0.019
miR-532-3p	24.9 (22.8, 27.6)	28.4 (24.8, 31.8)	0.071
miR-532-5p	28.4 (26.2, 30.7)	27.7 (24.8, 30.2)	0.5
miR-126-3p	50 (39, 53)	55 (45, 59)	0.073
miR-126-5p	36.0 (34.6, 40.1)	36.2 (32.6, 39.7)	0.7

^1^ n (%); Median (Q1, Q3); ^2^ Pearson’s Chi-squared test; Wilcoxon rank sum test; Wilcoxon rank sum exact test; ^3^ maximal stenosis of extracranial carotid arteries; M—male; DM—type 2 diabetes mellitus; BMI—body-mass index; RBC—red blood cells; WBC—white blood cells; LDL—low-density lipoprotein cholesterol.

**Table 3:** Subanalysis of the ECAS group with regards to smoking status.

	Non-Smokers (n = 13) ^1^	Smokers (n = 10) ^1^	*p*-Value ^2^
Stroke	3 (23%)	6 (60%)	0.10
M	2 (15%)	7 (70%)	0.013
Age, years	69 (64, 72)	62 (49, 69)	0.067
LDL-C, mmol/L	2.34 (1.44, 3.49)	1.53 (1.37, 2.34)	0.2
BMI, kg/m^2^	26.0 (24.4, 27.0)	26.5 (25.9, 34.0)	0.5
DM	5 (38%)	3 (30%)	>0.9
miR-712-5p	23.8 (22.4, 27.6)	26.4 (20.5, 29.6)	0.8
miR-712-3p	30.2 (29.9, 30.5)	30.2 (27.7, 30.6)	>0.9
miR-106b-3p	31.71 (30.38, 33.74)	31.13 (26.76, 31.78)	0.3
miR-106b-5p	29.98 (29.64, 30.23)	30.09 (29.91, 30.26)	0.4
miR-146a-3p	26.0 (25.0, 28.1)	27.2 (23.6, 28.7)	0.8
miR-146a-5p	28.13 (26.19, 28.63)	29.18 (28.58, 29.68)	0.15
miR-100-3p	28.8 (23.7, 31.3)	28.9 (27.4, 29.8)	0.8
miR-100-5p	26.3 (23.3, 28.5)	30.3 (23.3, 31.3)	0.3
miR-200c-3p	29.32 (28.56, 30.51)	29.54 (27.68, 29.72)	0.6
miR-200c-5p	25.3 (24.6, 28.2)	27.5 (24.6, 31.7)	0.7
miR-494-3p	32.7 (26.8, 38.5)	32.0 (29.6, 33.9)	>0.9
miR-494-5p	35 (31, 41)	36 (32, 42)	>0.9
miR-532-3p	23.62 (22.49, 26.67)	25.45 (24.01, 28.48)	0.2
miR-532-5p	28.2 (26.3, 30.7)	28.8 (26.2, 30.7)	>0.9
miR-126-3p	50 (43, 53)	47 (39, 52)	0.8
miR-126-5p	36.7 (35.0, 39.4)	35.3 (33.9, 40.5)	0.8

^1^ n (%); Median (Q1, Q3). ^2^ Fisher’s exact test; Wilcoxon rank sum test; Wilcoxon rank sum exact test.

**Table 4:** Major clinical characteristics of the ICAS patient cohort.

(ID) Age/Gender	Stroke Status	Clinical Characteristics	ICAS	ECAS	№ of Aff. Cerebr. Art.
(1) 58/M	Asymptomatic	Smoking, dyslipidemia, AH	60–65% (left ICA)	Occlusion (right ICA), 60–65% (left ICA), 20–25% (left VA)	4
(2) 57/M	Asymptomatic	Smoking, AH	60–70% (ICA bilaterally)	50% (left ICA), 25% (right VA), 60–70% (left VA)	5
(3) 60/M	Asymptomatic	Dyslipidemia, AH	50% (left ICA) 60% (left MCA), 70% (PCA bilateral), 50% (BA)	90% (left ICA), 60% (left CCA), 15% (right ICA), 75% (right VA), 65% (left VA)	10
(4) 66/F	Symptomatic	AH	70% (right MCA)	50% (right ICA)	2
(5) 61/M	Asymptomatic	DM, smoking, AH	50–55% (ICA bilateral), 95% (left VA), 95% (BA)	75–80% (right CCA), 45% (right ICA), 25% (left CCA), 50% (left ICA)	8
(6) 57/M	Symptomatic	DM, smoking, AH	45–50% (right ICA), 25–30% (left ICA)	50% (left ICA), 65–75% (right ICA), occlusion (left VA)	5
(7) 65/M	Asymptomatic	AH	70% (right MCA)	90% (right ICA), 70% (left ICA)	3
(8) 65/F	Symptomatic	AH	60% (left MCA)	30–35% (right ICA), 40–45% (left ICA)	3
(9) 60/M	Symptomatic	AH	20–25% (left ICA), 80% (right MCA)	25–30% (left VA), 35% (right ICA), 40% (left ICA)	5
(10) 69/M	Symptomatic	Smoking, dyslipidemia, AH	20% (BA), 50% (ICA bilateral), 70–75% (right MCA), 50% (left ACA), 60% (right VA), 30% (left VA)	65% (right ICA), 50% (left ICA), 45% (right CCA), 50% (left CCA), 35% (right VA), 25% (left VA)	13
(11) 68/M	Symptomatic	DM, dyslipidemia, AH	40–45% (ICA bilaterally)	60% (left ICA), 50% (right ICA), 25% (right CCA), 15% (left CCA)	6
(12) 70/M	Asymptomatic	AH	30–40% (ICA bilateraly)	70% (right ICA), 30–40% (both VA)	5

Notes: ICA—internal carotid artery; CCA—common carotid artery; MCA—middle cerebral artery; PCA—posterior cerebral artery; ACA—anterior cerebral artery; VA—vertebral artery; BA—basilar artery; AH—arterial hypertension; DM—type 2 diabetes mellitus; M—male; F—female; ICAS—intracranial atherosclerosis; ECAS—extracranial atherosclerosis. ‘Symptomatic’ denotes ischemic stroke, ipsilateral to the intracranial artery with the greatest degree of stenosis.

## Data Availability

The data that support the findings of this study are available from the corresponding author, P.K., upon reasonable request.

## Supplementary Materials

The following supporting information can be downloaded at: https://www.mdpi.com/article/10.3390/biomedicines13020514/s1, Figure S1. Visualization of variable importance in a multivariable logistic regression model regression for intracranial atherosclerosis as an ‘outcome’ variable (see ‘Statistics’ section): the more positive each variable lies on both charts the more important it is in the model; Table S1. Univariable logistic regression for intracranial atherosclerosis occurrence as dependent variable; Table S2. Association of variables of interest with smoking status (using ‘Smoking’ as dependent variable in a logistic regression model).
