# Supplementary material for: Differential Pattern of Circulating MicroRNA Expression in Patients with Intracranial Atherosclerosis
Source: Biomedicines. 2025 Feb 19;13(2):514. doi: 10.3390/biomedicines13020514 (PMC11853257; doi:10.3390/biomedicines13020514)

**Supplementary Figure S1.** Visualization of variable importance in a multivariable logistic regression model regression for intracranial atherosclerosis as an 'outcome' variable (see 'Statistics' section): the more positive each variable lies on both charts the more important it is in the model.

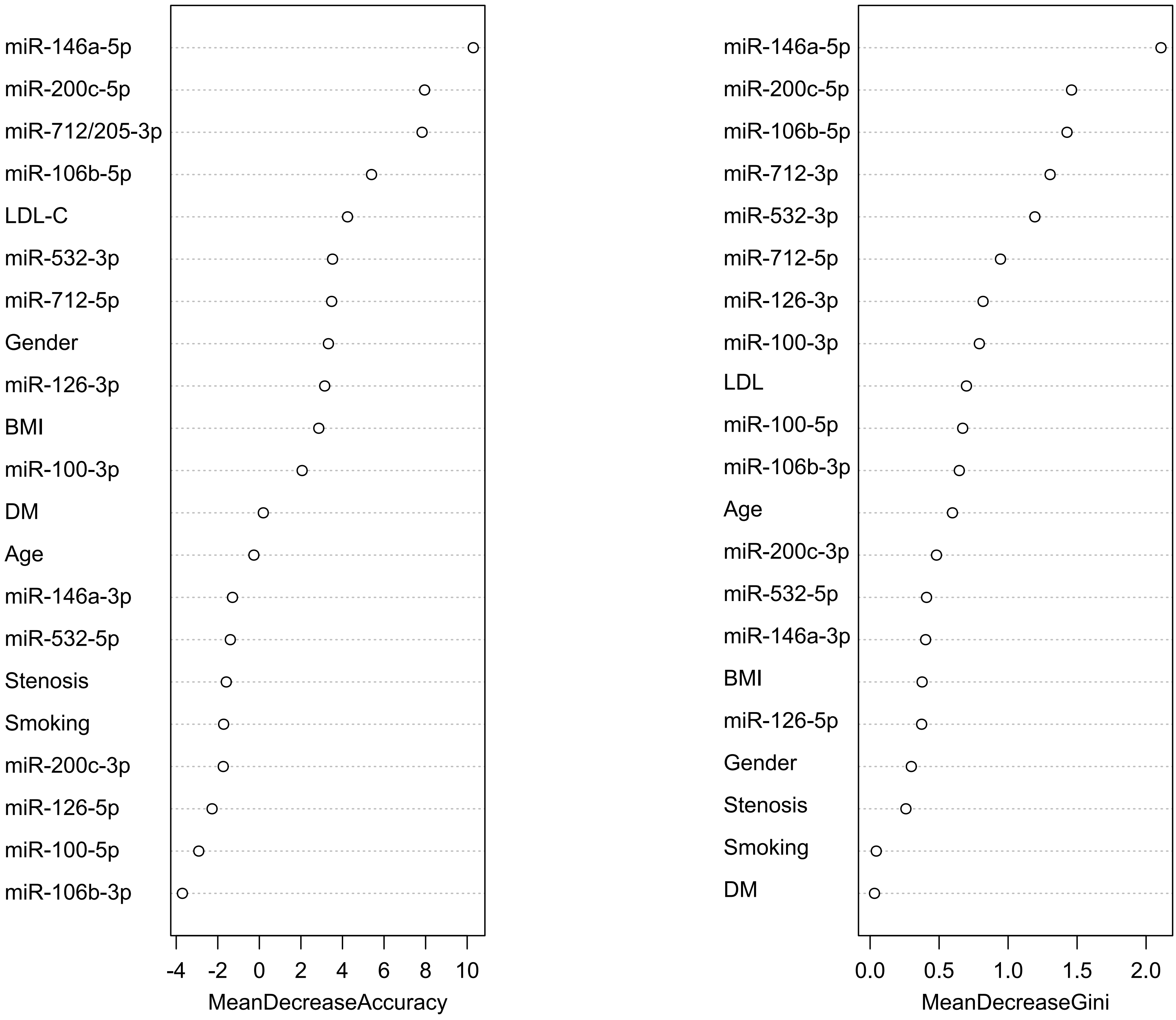

Supplement: Supplementary file 1 [file biomedicines-13-00514-s001.zip › Supplementary Figure S1.pdf]
