# Supplementary material for: Differential Pattern of Circulating MicroRNA Expression in Patients with Intracranial Atherosclerosis
Source: Biomedicines. 2025 Feb 19;13(2):514. doi: 10.3390/biomedicines13020514 (PMC11853257; doi:10.3390/biomedicines13020514)
Supplement: Supplementary file 1 [file biomedicines-13-00514-s001.zip › Supplementary Table S1.pdf]

Supplementary Table S1. Univariable logistic regression for intracranial atherosclerosis occurrence as dependent variable

Characteristic	N	OR[1]	95% CI[1]	p-value
Gender	35			
F		1.00	—	
M		7.78	1.59, 58.9	0.020
Age, years	35	0.96	0.89, 1.04	0.3
LDL-C, mmol/l	35	0.67	0.27, 1.47	0.3
Smoking	35			
No		1.00	—	
Yes		0.93	0.22, 3.82	>0.9
BMI, kg/m^2	35	0.93	0.74, 1.14	0.5
DM	35			
No		1.00	—	
Yes		0.94	0.20, 4.03	>0.9
Carotid stenosis	35	1.02	0.98, 1.08	0.4
miR-712-5p	35	1.19	1.03, 1.42	0.024
miR-712-3p	35	0.86	0.66, 1.07	0.2
miR-106b-3p	35	1.12	0.88, 1.45	0.4
miR-106b-5p	35	3.47	1.24, 15.0	0.055
miR-146a-3p	35	0.98	0.79, 1.22	0.9
miR-146a-5p	35	2.12	1.22, 4.90	0.033
miR-100-3p	35	0.86	0.71, 1.00	0.076
miR-100-5p	35	1.04	0.92, 1.18	0.5
miR-200c-3p	35	1.17	0.95, 1.51	0.2
miR-200c-5p	35	1.24	1.05, 1.52	0.022
miR-532-3p	35	1.16	0.99, 1.42	0.090
miR-532-5p	35	0.93	0.77, 1.10	0.4
miR-126-3p	35	1.09	0.99, 1.22	0.10
miR-126-5p	35	0.96	0.82, 1.09	0.6

[1] OR = Odds Ratio, CI = Confidence Interval
