# Supplementary material for: Differential Pattern of Circulating MicroRNA Expression in Patients with Intracranial Atherosclerosis
Source: Biomedicines. 2025 Feb 19;13(2):514. doi: 10.3390/biomedicines13020514 (PMC11853257; doi:10.3390/biomedicines13020514)
Supplement: Supplementary file 1 [file biomedicines-13-00514-s001.zip › Supplementary Table S2.pdf]

Supplementary Table S2. Association of variables of interest with smoking status (using 'Smoking' as dependent variable in a logistic regression model)

Characteristic	N	OR[1]	95% CI[1]	p-value
Gender	35			
F		1.00	—	
M		7.43	1.70, 41.6	0.012
ICAS	35			
No		1.00	—	
Yes		0.93	0.22, 3.82	>0.9
Age, years	35	0.90	0.80, 0.98	0.039
Stroke	35			
No		1.00	—	
Yes		2.12	0.55, 8.68	0.3
LDL-C, mmol/l	35	0.80	0.36, 1.67	0.6
BMI, kg/m^2	35	1.13	0.94, 1.41	0.2
DM	35			
No		1.00	—	
Yes		0.93	0.22, 3.82	>0.9
Stenosis	35	0.98	0.94, 1.01	0.3
miR-712-5p	35	0.97	0.85, 1.10	0.6
miR-712-3p	35	0.97	0.78, 1.20	0.8
miR-106b-3p	35	0.89	0.71, 1.11	0.3
miR-106b-5p	35	1.28	0.73, 2.74	0.4
miR-146a-3p	35	0.98	0.79, 1.21	0.8

Characteristic	N	OR[1]	95% CI[1]	p-value
miR-146a-5p	35	1.07	0.89, 1.36	0.5
miR-100-3p	35	1.00	0.87, 1.15	>0.9
miR-100-5p	35	1.04	0.92, 1.17	0.6
miR-200c-3p	35	0.98	0.78, 1.20	0.8
miR-200c-5p	35	1.03	0.91, 1.17	0.6
miR-494-3p	35	1.01	0.88, 1.15	0.9
miR-494-5p	35	0.96	0.87, 1.06	0.4
miR-532-3p	35	1.06	0.92, 1.25	0.4
miR-532-5p	35	1.01	0.86, 1.19	>0.9
miR-126-3p	35	0.95	0.86, 1.04	0.3
miR-126-5p	35	1.05	0.93, 1.22	0.4

[1] OR = Odds Ratio, CI = Confidence Interval
